# Disruption of cellular calcium homeostasis by duck Tembusu virus facilitates viral replication via AMPK pathway activation

**DOI:** 10.3389/fcimb.2026.1743907

**Published:** 2026-02-09

**Authors:** Ziding Yu, Xiaoyong Chen, Wenxiao Zhuo, Zhenxing Ma, Zejun Xu, Kuanhui Liu, Julong Wang, Ting Li, Guangshuang Zhu, Benzhong Wang, Ran Xiong, Chao Li, Haiyan Zhang, Jingliang Su

**Affiliations:** 1 Wuhu Vocational Technical University, Wuhu, China; 2 Detection of Food-borne Pathogenic Microorganisms Engineering Research Center of Wuhu, Wuhu, China; 3 Xingzhi College, Zhejiang Normal University, Jinhua, China; 4 College of Life Sciences, Zhejiang Normal University, Jinhua, China; 5 Key Laboratory of Animal Epidemiology and Zoonosis, Ministry of Agriculture, College of Veterinary Medicine, China Agricultural University, Beijing, China

**Keywords:** AMPK, antiviral target, Ca2+ homeostasis, DTMUV, VGCCs, viral RNA replication

## Abstract

**Background:**

The flavivirus Duck Tembusu virus (DTMUV) exhibits high pathogenicity and transmissibility, posing a severe threat to the poultry industry in China and Southeast Asia. Although the molecular mechanisms of DTMUV pathogenesis remain unclear, its potential to disrupt intracellular calcium ion (Ca²^+^) homeostasis, a known driver of viral replication, has not been investigated. Here, we investigated the role and underlying mechanism of Ca²^+^ homeostasis in DTMUV infection.

**Methods:**

Fluo-4AM staining and flow cytometry of duck embryo fibroblasts (DEFs) were used to detect cytoplasmic Ca²^+^. To modulate Ca²^+^ levels and AMP-activated protein kinase (AMPK) activity, we used voltage-gated calcium channel (VGCC) blockers (verapamil, diltiazem hydrochloride), a Ca²^+^ chelator (BAPTA-AM), and an AMPK inhibitor (Compound C). Viral entry, genomic RNA replication (targeting the DTMUV NS5 gene), viral yield, and release were evaluated via qRT-PCR and plaque assays. AMPK activation was detected using western blotting with anti-phospho-AMPKα (Thr172) and anti-AMPKα antibodies. Statistical analyses were performed using Student’s t-test or two-way analysis of variance.

**Results:**

DTMUV infection significantly increased cytoplasmic Ca²^+^ in DEFs at 6, 8, 10, and 12 hours post-infection. This elevation was suppressed by treatment with verapamil or diltiazem hydrochloride, indicating that DTMUV induces extracellular Ca²^+^ influx via VGCCs. Functional assays showed that reducing cytoplasmic Ca²^+^ via treatment with VGCC blockers or BAPTA-AM specifically inhibited DTMUV RNA replication, but not viral entry or release, decreasing progeny virus production. Further mechanistic analysis revealed that DTMUV infection activates the AMPK pathway in a Ca²^+^-dependent manner, with Compound C-mediated AMPK inhibition dose-dependently suppressing viral RNA replication and progeny yield.

**Conclusions:**

DTMUV disrupts host Ca²^+^ homeostasis to activate AMPK, which promotes viral RNA replication. This study provides novel insights into DTMUV pathogenesis and identifies Ca²^+^–AMPK signaling as a potential anti-DTMUV target.

## 1 Introduction

Duck Tembusu virus (DTMUV) is an enveloped, single-stranded positive-sense RNA virus belonging to the genus Flavivirus within the family Flaviviridae. The genome of DTMUV is approximately 11,000 nucleotides (nt) in length, containing one open reading frame (ORF) that encodes three structural proteins (C, PrM, E) and seven non-structural (NS) proteins (NS1, NS2A, NS2B, NS3, NS4A, NS4B, and NS5) (Tang et al., 2012). The Tembusu virus (TMUV) prototype strain MM1775 was first isolated from mosquitoes in 1955 (Platt et al., 2016). In 2010, a DTMUV outbreak occurred in southeastern China, characterized by encephalitis and a sharp decline in egg production in duck and goose flocks (Qiu et al., 2011). Subsequently, diseases associated with this virus were also reported in Southeast Asian countries (Homonnay et al., 2014; Ninvilai et al., 2018). In recent years, accumulating studies have confirmed that several DTMUV strains, characterized by substantial antigenic variation and belonging to a unique genetic cluster, have begun circulating in chicken flocks (Yu et al., 2021; Yan et al., 2022). DTMUV exhibits high pathogenicity and transmissibility, with infection in avian hosts leading to a marked decline in egg production and triggering neurological symptoms, typically encephalitis. Currently, this virus is widely distributed across China and Southeast Asia, causing enormous economic losses to the regional poultry industry. Although vaccination has historically been a key strategy for DTMUV control, effective prevention requires elucidating its pathogenic mechanisms to identify potential antiviral targets.

The calcium ion (Ca²^+^) is one of the most ubiquitous and versatile signaling molecules in eukaryotic cells. As an intracellular second messenger, Ca²^+^ is involved in regulating almost all cellular physiological processes, including gene transcription, differentiation, proliferation, and kinase activation (Berridge et al., 2000, 2003). Under normal cellular physiology, intracellular Ca²^+^ homeostasis—maintaining stable cytoplasmic Ca²^+^ concentrations—is tightly regulated by the coordinated interplay of channels, transporters, and pumps (Berridge et al., 2003). As a stimulant, viruses often disrupt intracellular Ca²^+^ homeostasis by hijacking Ca²^+^-mediated signaling transduction pathways to facilitate the infection cycle. For instance, upon infecting host cells, duck enteritis virus (DEV) and porcine reproductive and respiratory syndrome virus (PRRSV) increase cytoplasmic Ca²^+^ concentrations, inducing autophagy mediated by the calmodulin-dependent protein kinase II (CaMKK-II) and adenosine 5′-monophosphate-activated protein kinase (AMPK) pathway to promote their own replication (Yin et al., 2018; Manicassamy et al., 2023). Within the genus Flavivirus, host cell infection by West Nile virus (WNV) and dengue virus (DENV) also induces an increase in cytoplasmic Ca²^+^ concentration. Importantly, pharmacological inhibition of this Ca²^+^ elevation significantly reduces the production of viral particles, suggesting that the infection-induced elevation in cytoplasmic Ca²^+^ is essential for the replication of these flaviviruses (Scherbik and Brinton, 2010; Dionicio et al., 2018).

Despite the well-documented association between Ca²^+^ homeostasis and flavivirus infection, its role in DTMUV infection remains uncharacterized. Here, we report the first investigation of this association, demonstrating that DTMUV infection significantly increases cytoplasmic Ca²^+^ concentrations in host cells. Furthermore, this elevation in Ca²^+^ activates the AMPK signaling pathway, a critical mediator of DTMUV RNA replication. These findings lay a solid foundation for elucidating the molecular mechanisms of DTMUV pathogenesis and offer new insights into potential targets for anti-DTMUV strategies.

## 2 Materials and methods

### 2.1 Cell culture and virus

Baby hamster kidney (BHK)-21 cells were obtained from the American Type Culture Collection (Manassas, VA, USA). Duck embryo fibroblasts (DEFs) were prepared from 10-day-old duck embryos obtained from Shandong Health-Tech Laboratory Animal Breeding Co., Ltd. (Jinan, China) following standard procedures. DEFs cells were cultured in Dulbecco’s modified Eagle’s medium (DMEM) containing 10% fetal bovine serum (FBS) and 1% penicillin-streptomycin at 37 °C with 5% CO2. DTMUV strain JXSP was isolated and preserved in our laboratory.

### 2.2 Drug treatment and virus infection

The calcium channel blockers verapamil (Cat# HY-14275) and diltiazem hydrochloride (Cat# HY-14656), the AMPK inhibitor Compound C (Cat# HY-13418A), and calcium chelating agent BAPTA-AM (Cat# HY-100545) were purchased from MedChemExpress (Monmouth Junction, NJ, USA). All drugs were dissolved and stored in DMSO according to manufacturer instructions. Prior to use, each drug was diluted to its final working concentration in DMEM containing 1% FBS and 1% penicillin-streptomycin: verapamil (25 μM), diltiazem hydrochloride (50 μM), BAPTA-A (25 μM), and Compound C (2.5 μM or 5 μM). For drug treatment and virus infection, DEFs grown in 6-well plates or 12-well plates were washed thrice with sterile phosphate-buffered saline (PBS), then inoculated with DTMUV (multiplicity of infection [MOI] = 1) concurrent with the corresponding drug treatment. After 1 hour, the supernatant was replaced with fresh drug-containing DMEM. Cells and supernatants were collected at the indicated time points for reverse transcription quantitative real-time PCR (RT-qPCR), western blotting, or virus titration analysis.

To assay viral entry, DEFs cultured in 6-well plates were washed thrice with sterile PBS and pretreated with diltiazem hydrochloride (50 μM) and BAPTA-AM (25 μM) for 1 hour. After another three washes with sterile PBS, cells were infected with DTMUV (MOI = 1) at 4°C for 1 hour to allow viral attachment while blocking fusion. After a wash to remove unbound virus, cells were shifted to 37°C for 1 hour to initiate fusion. Viral RNA levels in the cytoplasm were quantified by RT-qPCR at 2 hours post-infection (hpi).

To assay viral replication, DEFs cultured in 6-well plates were washed thrice with sterile PBS and incubated with DTMUV for 2 hours. The supernatants were removed, and the cells were cultured in fresh medium containing diltiazem hydrochloride (50 μM) or BAPTA-AM (25 μM). At 6 hpi, the infected cells were collected and expression of the DTMUV NS5 gene was quantified by RT-qPCR.

To assay viral release, DEFs cultured in 6-well plates were washed thrice with sterile PBS and incubated with DTMUV. At 10 hpi, the supernatants were replaced with fresh medium containing diltiazem hydrochloride (50 μM) or BAPTA-AM (25 μM). Two hours later, the cell supernatants were harvested and titrated by plaque assay.

Plaque assay.

Cell supernatant samples were serially diluted 10-fold with DMEM containing 2% FBS and 1% penicillin-streptomycin. Each dilution was used to inoculate a monolayer of BHK-21 cells in 12-well plates (1 mL/well), followed by incubation at 37°C for 1 hour with gentle rocking every 15 min. After removing the inoculum, cells were washed thrice with sterile PBS and overlaid with DMEM containing 1% (wt/vol) low-melting-point agar (Amresco, Spokane, WA, USA), 2% FBS, and 1% penicillin-streptomycin. Following overlay solidification at 4°C, the plates were incubated at 37°C for 3 days. Plaques were visualized and counted following staining of viable cells with neutral red (0.02%).

### 2.3 Viral RNA extraction and RT-qPCR

Total RNA of virus-infected DEFs was extracted using TRIzol reagent. The cDNA was synthesized using random primers and a reverse transcription system (Promega, Madison, WI, USA), in accordance with the manufacturer’s instructions. RT-qPCR was performed on a Bio-Rad CFX Connect instrument using the SYBR green SuperReal PreMix Plus kit (Tiangen, Beijing, China) and the following JXSP NS5-specific primers: forward, 5’-TTGGGACACCTTGCAAAACG-3’; reverse, 5’-TTGCCACGATGTTCATGACC-3’, and duck GAPDH-specific primers: forward, 5’- AAATTGTCAGCAATGCCTCTTG-3’; reverse, 5’-TGGCATGGACAGTGGTCATAA-3’. The 20-μL reaction mixture comprised 10 μL SuperReal PreMix Plus, 0.3 μL of specific primers, 7.7 μL sterile double-distilled water and 2 μL of cDNA template. The cycling conditions were as follows: initial denaturation at 95°C for 15 min; and 40 cycles of denaturation at 95°C for 10 s and annealing/extension at 60 °C for 30 s. GAPDH was used as the reference housekeeping gene for internal standardization. The data of quantitative real-time PCR analysis were shown in normalized ratios and auto-calculated using ΔΔCT method.

### 2.4 Measurement of cytoplasmic Ca^2+^ concentration

Cytoplasmic Ca2+ concentrations were measured using Fluo-4AM (Beyotime, Shanghai, China) and flow cytometry. Briefly, DEFs infected with DTMUV at different time points or treated with different drugs were rinsed three times with sterile PBS, followed by staining with Fluo-4AM (diluted to 5 µM in sterile PBS) at 37 °C for 1 h and another three rinses. The cells were then detached via trypsinization, filtered through a 40-μm cell strainer (Beyotime, Shanghai, China) to prepare a single-cell suspension, and analyzed on a LSR II flow cytometer (BD Biosciences). The mean fluorescence intensity (MFI) analysis was performed using FlowJo software (Version 10.8.1, BD Biosciences, San Jose, CA, USA) according to the following standardized protocol. Raw FCS files of the samples were imported into FlowJo. Cell debris and doublets were excluded by gating to retain target cells, with consistent gating applied across all samples. For each assay, 10,000 target cells were collected within this gate. In the FITC channel, the MFI of the gated target population was quantified using the built-in Mean function of FlowJo for Fluo-4AM-stained samples. Statistical analysis of the measured MFI values was conducted using GraphPad Prism 8 (see Data Analysis).

### 2.5 Western blotting

DEFs were lysed using cell lysis buffer containing 1% phenylmethanesulfonyl fluoride (Beyotime), following the manufacturer’s protocol. Cell fragments were removed by low-temperature centrifugation (4°C, 12,000 × g, 10 min). The resulting supernatant was thoroughly mixed with 6× sodium dodecyl sulfate-polyacrylamide gel electrophoresis (SDS-PAGE) sample loading buffer (Beyotime), boiled for 5 min, separated by SDS-PAGE, and transferred onto a formaldehyde-activated polyvinylidene fluoride (PVDF) membrane. The PVDF membrane was blocked with 5% skim milk and probed with the following antibodies: anti-phospho-AMPKα (Thr172) antibody (Cell Signaling Technology, Beverly, MA, USA), to assess AMPK activation levels; anti-AMPKα antibody (Cell Signaling Technology), to measure total AMPK protein expression; and anti-β-actin antibody (Beyotime), as a control for sample consistency.

### 2.6 Data analysis

Data were processed using GraphPad Prism 8 (GraphPad Software Inc., La Jolla, CA, USA). Statistical comparisons between two groups were performed using Student’s t-test (for two-group comparisons) or one-way/two-way analysis of variance (ANOVA) with multiple comparisons, as appropriate. A p value < 0.05 was considered statistically significant.

## 3 Results

### 3.1 DTMUV infection disrupts cytoplasmic Ca^2+^ homeostasis by inducing extracellular Ca²^+^ influx through voltage-gated calcium channels

**Figure 1:**
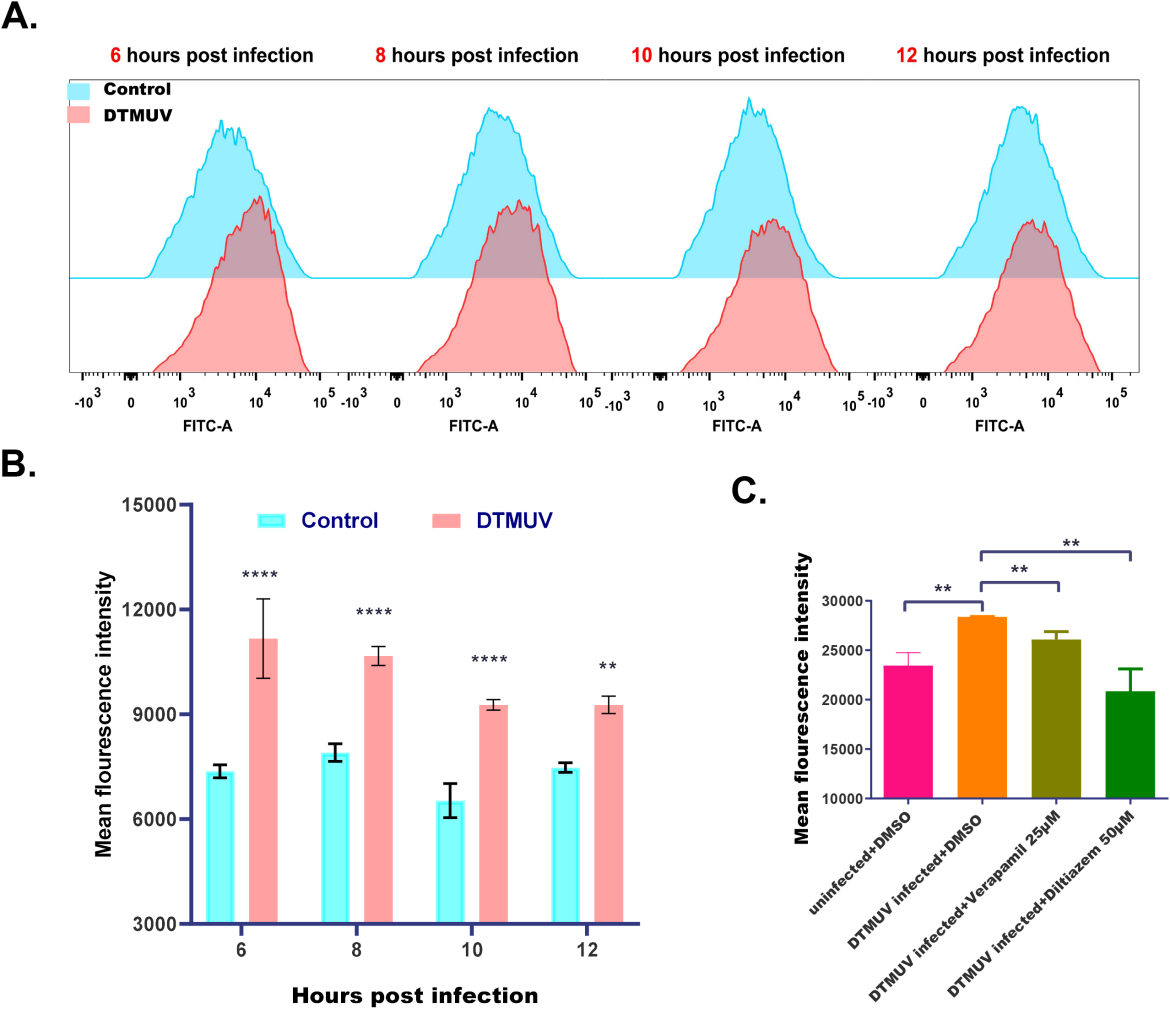
DTMUV infection increases cytoplasmic Ca^2+^ levels in DEFs. **(A)** Flow cytometry profiles showing cytoplasmic Ca2+ levels in DEFs with and without DTMUV infection and probed with Flou-4AM. **(B)** Cytoplasmic Ca2+ levels of DEFs with (red) and without (blue) DTMUV infection (MOI = 0.1) for 6, 8, 10, or 12 hours, expressed as mean fluorescence intensity (MFI). **(C)** Cytoplasmic Ca2+ levels of DEFs with and without DTMUV infection and concurrent treatment with DMSO (control) verapamil or diltiazem hydrochloride. Data expressed as mean ± standard deviation (n = 3), analyzed using Student’s t-test; *p < 0.05, **p < 0.01, ****P<0.0001.

Mounting evidence from virology studies has established that many viral pathogens disrupt intracellular Ca²^+^ homeostasis to create a favorable microenvironment for their propagation. To determine whether DTMUV infection affects this process, we monitored cytoplasmic Ca²^+^ concentrations throughout the infection cycle. Cytoplasmic Ca²^+^ levels were quantified by flow cytometry as MFI following staining with Fluo-4AM, a Ca²^+^-specific fluorescent probe. As illustrated in **Figures 1A and B**, intracellular Ca²^+^ levels of DEFs significantly increased at 6, 8, 10, and 12 hpi. This elevation was significantly inhibited by concurrent treatment with VGCC blockers (verapamil or diltiazem hydrochloride) (**Figure 1C**), indicating that DTMUV-induced cytoplasmic Ca²^+^ influx is primarily mediated through VGCCs. Collectively, these findings demonstrated that DTMUV infection disrupts Ca²^+^ homeostasis in host cells.

### 3.2 Cytoplasmic Ca²^+^ is an essential regulator of DTMUV production in DEFs

**Figure 2:**
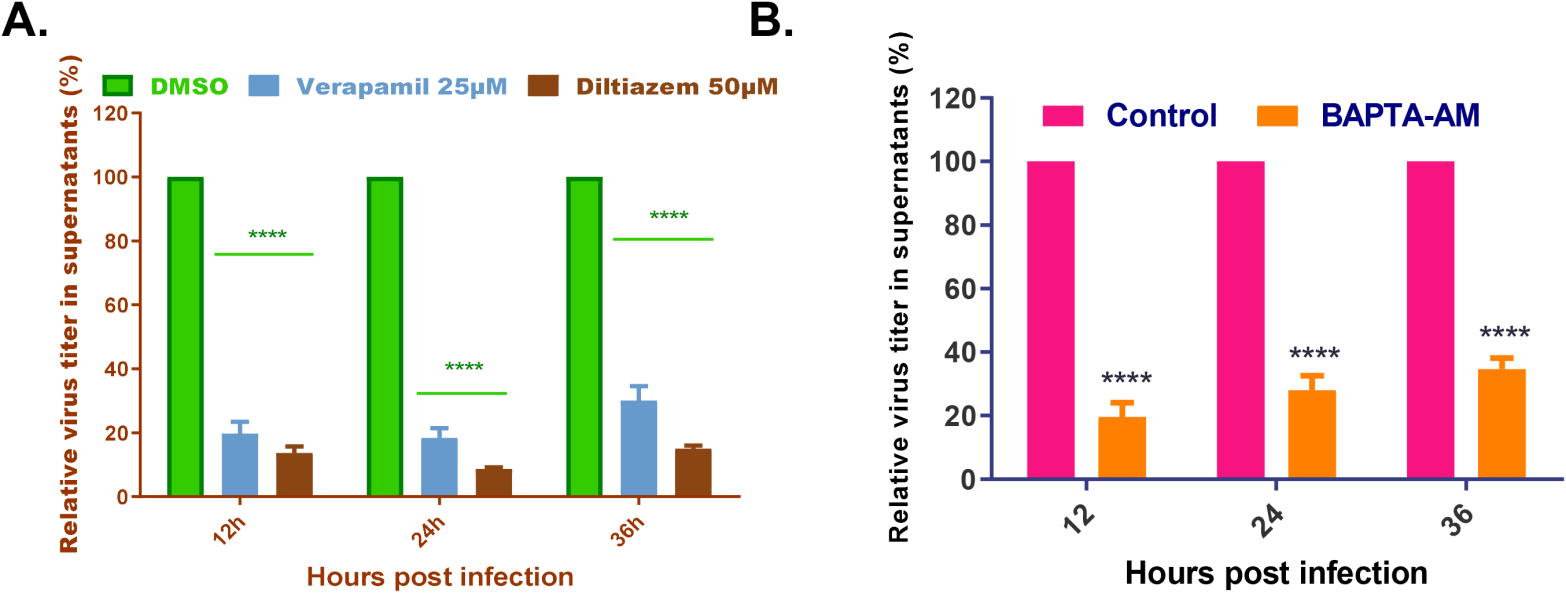
VDCC blockers and a cytoplasmic Ca^2+^ chelator reduce DTMUV particle production. **(A, B)** Analysis of plaque assays of DEFs infected with DTMUV and treated with verapamil (25 µM), diltiazem hydrochloride (50 µM), or DMSO (control; **(A)**), and BAPTA-AM (25 µM) or DMSO (control; **(B)**). Results expressed as the viral titer ratio (%) between each drug-treated group and the control group at 12, 24, and 36 hpi. Data expressed as mean ± standard deviation of triplicate samples, analyzed by two-way ANOVA with multiple comparisons. *p < 0.05, **p <0.01, ***p <0.001, ****p < 0.0001. Results shown are representative of three independent experiments.

Given the functional link between viral-induced dysregulation of Ca²^+^ homeostasis and viral replication, we sought to determine whether the DTMUV-mediated elevation in cytoplasmic Ca²^+^ contributes to viral propagation. Verapamil and diltiazem hydrochloride were initially employed to inhibit extracellular Ca^2+^ influx and reduce cytoplasmic Ca^2+^ levels, followed by measurement of progeny virus production. Both VGCC blockers significantly reduced the number of progeny virus particles in DEFs with diltiazem hydrochloride exhibiting the most pronounced inhibitory effect (**Figure 2A**). Additionally, we evaluated DTMUV production in the presence of the cytoplasmic Ca^2+^ chelator BAPTA-AM. As shown in **Figure 2B**, replication of DTMUV in DEFs was markedly diminished when either extracellular or cytoplasmic Ca^2+^ was chelated. These findings indicated that DTMUV promotes its replication by elevating cytoplasmic Ca^2+^ levels.

### 3.3 Reducing cytoplasmic Ca2+ inhibits the replication step of DTMUV infection

To further delineate the specific step(s) in the DTMUV life cycle that are regulated by cytoplasmic Ca²^+^, we performed a series of stage-specific functional assays of viral entry, viral RNA replication, and viral release.

**Figure 3:**
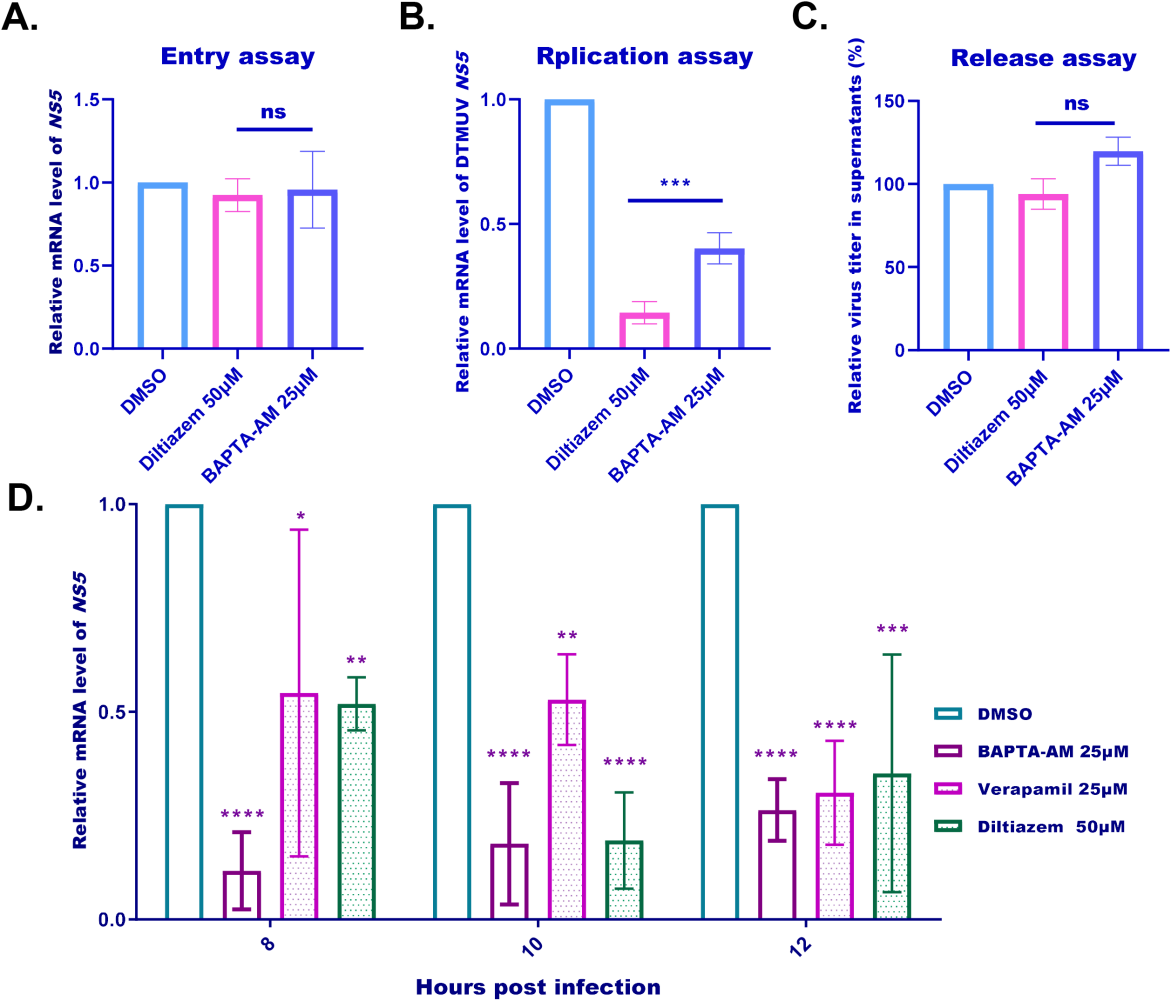
VDCC blockers and a cytoplasmic Ca^2+^ chelator inhibit the replication step of DTMUV infection. **(A)** Viral entry assay of DEFs pretreated with DMSO, diltiazem (50 µM), or BAPTA-AM (25 µM) for 1 hour prior to DTMUV infection (MOI = 1) at 4°C for 1 hour and fusion at 37°C. Viral RNA levels in the cytoplasm were quantified by RT-qPCR at 2 hours post-infection (hpi), expressed as relative DTMUV mRNA levels between the drug-treated groups and the control group. **(B)** Viral replication assay of DEFs infected with DTMUV (MOI = 1) prior to treatment with DMSO (control), diltiazem hydrochloride (50 µM), or EAPTA-AM (25 µM) at 2 hpi, and RT-qPCR analysis of viral RNA replication in infected cells at 6 hpi, expressed as relative DTMUV mRNA levels between the drug-treated and control groups. **(C)** Plaque assay of viral release in DEFs cultured infected with DTMUV (MOI = 1) prior to treatment with DMSO (control), diltiazem hydrochloride (50 µM), or EAPTA-AM (25 µM) at 10 hpi and plating at 12 hpi. Results expressed as the viral titer ratio (%) between the drug-treated groups and the control group. **(D)** Viral replication assay of DEFs infected with DTMUV (MOI = 1) prior to treatment with DMSO (control) or alternative forms of verapamil (25 µM), diltiazem hydrochloride (50 µM), or BAPTA-AM (25 µM) at 1 hpi. Infected cells were harvested for RT-qPCR analysis of DTMUV mRNA levels at 8, 10, and 12 hpi, expressed as relative DTMUV mRNA levels between the drug-treated and control groups. Data expressed as mean ± standard deviation of triplicate samples, analyzed by one-way or two-way ANOVA with multiple comparisons; *p < 0.05, **p <0.01, ***p <0.001, ****p <0.0001. Data shown are representative of three independent experiments. ns: no significant difference.

For the viral entry assay (**Figure 3A**), DEFs were pretreated with diltiazem hydrochloride or BAPTA-AM prior to a brief DTMUV attachment and entry period. Intracellular viral RNA was quantified by RT-qPCR after removal of unbound virus. No significant difference was observed between the treated and untreated groups, indicating that reducing cytoplasmic Ca²^+^ does not affect the DTMUV entry step.

For the viral RNA replication assay (**Figure 3B**), DEFs were first infected with DTMUV to allow efficient viral entry, then treated with diltiazem hydrochloride or BAPTA-AM following the removal of unbound virus. At specified time points, intracellular viral RNA was quantified by RT-qPCR. In contrast to the viral entry results, reducing cytoplasmic Ca²^+^ levels with either drug significantly inhibited the accumulation of DTMUV genomic RNA, demonstrating a direct effect on viral RNA replication.

To assess viral release, DEFs were infected with DTMUV and treated with diltiazem hydrochloride or BAPTA-AM at 10 hpi. At 12 hpi, viral titers in the cell culture supernatant were quantified using plaque assay. As shown in **Figure 3C**, there was no significant difference in the ratio of released virus to intracellular virus between treated and untreated groups (p > 0.05), demonstrating that cytosolic Ca²^+^ reduction does not impact DTMUV release.

To validate the reproducibility and generalizability of these findings, we further investigated the effects of verapamil, diltiazem hydrochloride, and BAPTA-AM on DTMUV RNA replication throughout the infection cycle in DEFs and assessed their impact on DTMUV genomic RNA replication. Consistently, all three drugs also significantly inhibited the replication of DTMUV genomic RNA in these cells throughout the infection cycle (**Figure 3D**).

Collectively, these data demonstrated that reducing cytosolic Ca²^+^ levels specifically impairs the viral RNA replication step of the DTMUV life cycle.

### 3.4 Cytosolic Ca2+ promotes DTMUV replication by activating the AMPK pathway

Elevated cytosolic Ca²^+^ acts as a universal intracellular signal transducer regulating diverse cellular processes in eukaryotes, including activation of the AMPK pathway, a central regulator of energy homeostasis (Tokumitsu and Sakagami, 2022; Herzig and Shaw, 2017). Several viruses have been shown to actively exploit AMPK signaling to modulate their own life cycles, highlighting the critical role of this pathway in viral replication and pathogenesis (Gu et al., 2016; Yin et al., 2018; Zhang et al., 2023). Based on these observations, we hypothesized that intracellular Ca2+ promotes DTMUV replication via AMPK pathway activation.

**Figure 4:**
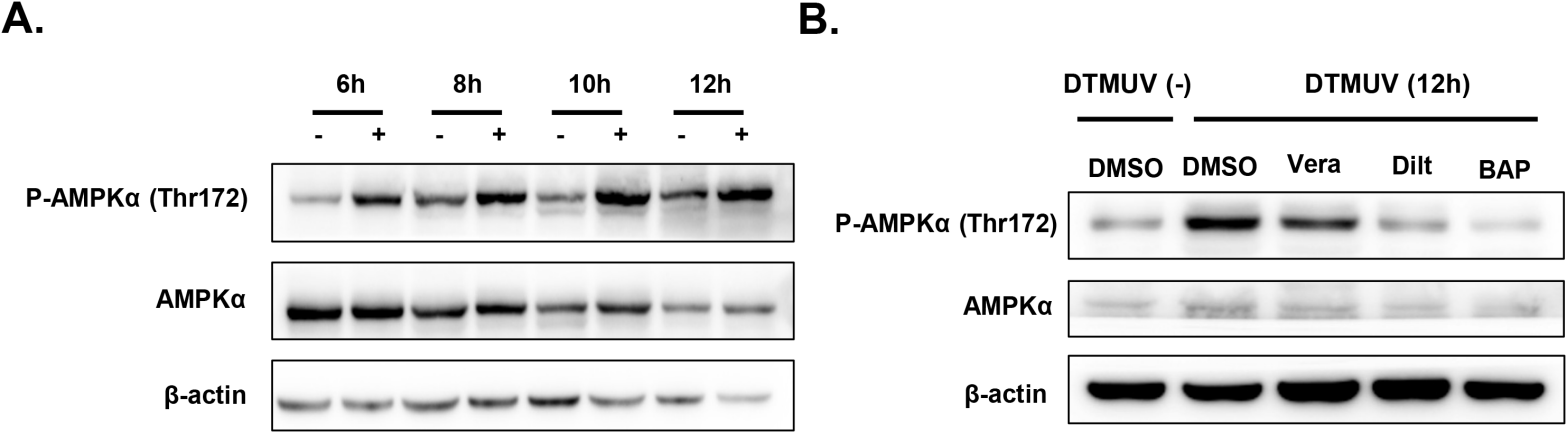
DTMUV-mediated AMPK activation is markedly diminished by treatment with VDCC blockers or a cytoplasmic Ca^2+^ chelator. **(A)** Western blotting of pAMPKα (Thr172) in DEFs infected with DTMUV (MOI = 1) and harvested at the indicated time points. **(B)** Immunoblotting analysis of pAMPKα (Thr172) levels in DEFs treated with DMSO (control), verapamil (25 µM; Vera), diltiazem hydrochloride (50 µM; Dilt), or BAPTA-AM (25 µM; BAP), with and without DTMUV infection (MOI = 1) for 12 hours.

To evaluate this, we first investigated the dynamic activation status of AMPK in DEFs following DTMUV infection. Compared with the non-infected group, AMPK activation was markedly enhanced at 6, 8, 10, and 12 hpi, as evidenced by a significant rise in phosphorylated (p)AMPK levels (**Figure 4A**). This finding confirmed that DTMUV infection induces AMPK pathway activation in DEFs.

Next, to determine whether DTMUV-mediated activation of AMPK signaling depends on cytosolic Ca²^+^ elevation, we treated cells with verapamil, diltiazem hydrochloride, or BAPTA-AM and observed significant suppression of AMPK activation with all three drugs (**Figure 4B**). These results clearly demonstrated that DTMUV infection induces AMPK activation in DEFs in a cytosolic Ca²^+^-dependent manner.

**Figure 5:**
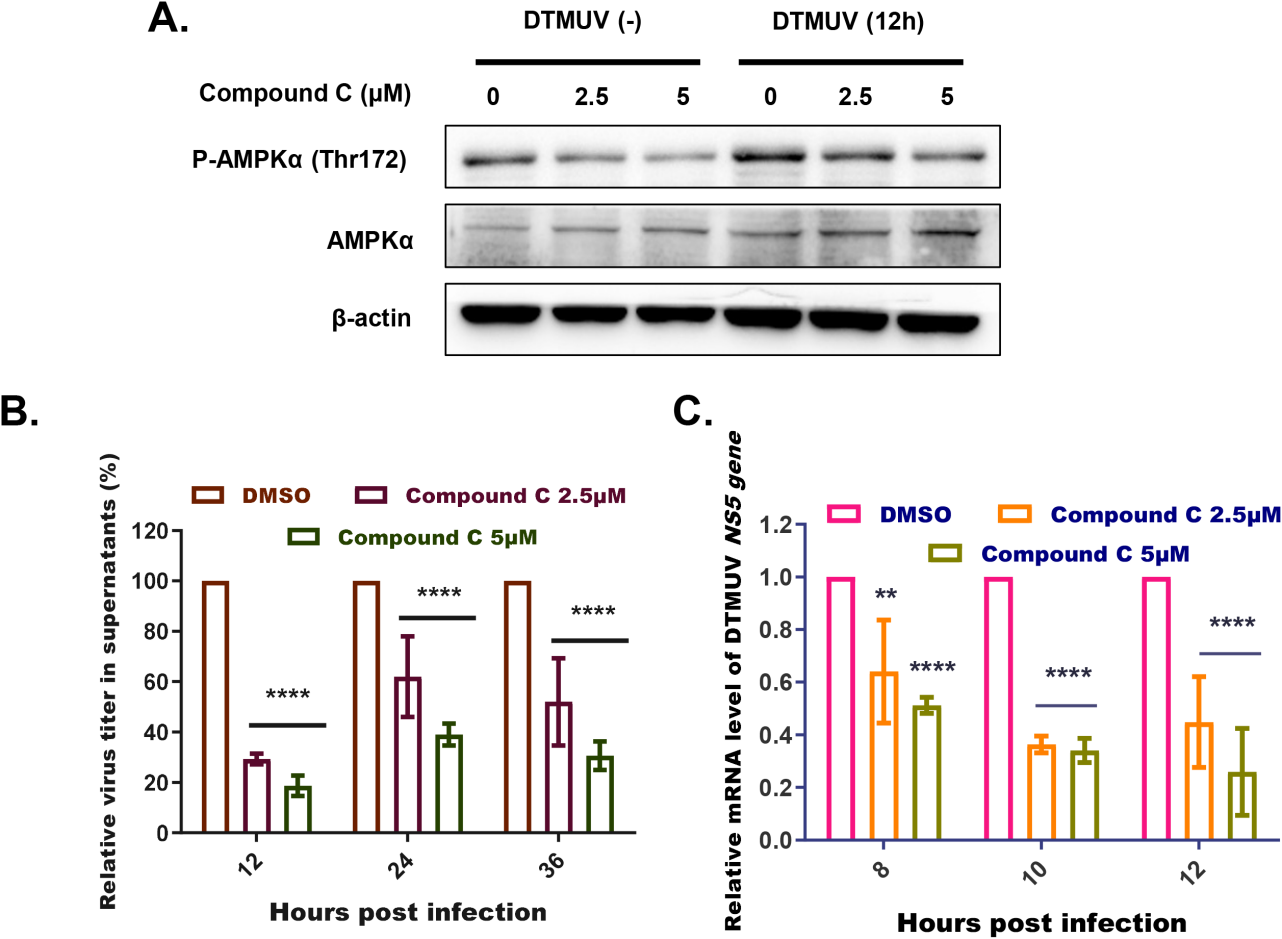
Inhibition of AMPK activation significantly reduces DTMUV replication. **(A)** Immunoblotting analysis of pAMPKα (Thr172) levels in DEFs treated with Compound C at a range of concentrations, with or without DTMUV infection (MOI = 1) for the indicated times. **(B)** Plaque assay of DTMUV production at 12, 24, and 36 hours post infection (hpi) in DEFs treated with DMSO (control) or Compound C (2.5 µM or 5 µM), expressed as the viral titer ratio (%) between the inhibitor-treated group and the control group. **(C)** RT-qPCR analysis of relative DTMUV mRNA levels at 8, 10, and 12 hpi in DEFs treated with Compound C (2.5 or 5 µM), expressed as the viral titer ratio (%) between the inhibitor-treated group and the control group. Data expressed as means ± standard deviation of triplicate samples, analyzed by two-way ANOVA with multiple comparisons; **p < 0.01, ****p < 0.0001. Results shown are representative of three independent experiments. ns: no significant difference.

To confirm that cytoplasmic Ca²^+^ promotes DTMUV replication through AMPK activation specifically, we treated infected DEFs with Compound C to pharmacologically suppress AMPK signaling. Western blot analysis verified that treatment with 2.5 µM or 5 µM Compound C dose-dependently reduced DTMUV-induced p-AMPKα levels without affecting total AMPKα (**Figure 5A**). Next, we assessed the impact of AMPK inhibition on DTMUV infection by quantifying both progeny virus production (**Figure 5B**) and viral RNA replication (**Figure 5C**). Compound C-mediated inhibition of DTMUV-induced AMPK activation significantly reduced both the yield of progeny virus particles and the accumulation of DTMUV genomic RNA in a dose-dependent manner.

## 4 Discussion

As a ubiquitous intracellular second messenger, Ca^2+^ plays a pivotal regulatory role in virtually all cellular processes throughout the cell cycle (Zhou et al., 2009). A growing body of evidence points to the active involvement of Ca^2+^ and Ca^2+^-related proteins in the viral life cycle, playing a critical role in viral pathogenesis (Casciano and Bouchard, 2018; Fujioka et al., 2018; Gu et al., 2016; Lee et al., 2024). In this study, we found that DTMUV infection disrupts Ca²^+^ homeostasis in host cells by increasing cytoplasmic Ca²^+^ levels. This elevation in cytoplasmic Ca²^+^ activates the AMPK pathway, promoting viral RNA replication and proliferation. To our knowledge, this is the first report linking Ca²^+^, the AMPK pathway, and DTMUV replication.

Under normal cellular physiological conditions, intracellular Ca²^+^ homeostasis—defined as the maintenance of a stable concentration of cytoplasmic free Ca²^+^—is tightly regulated by the coordinated interplay of multiple membrane transporters and regulatory proteins. These key mediators include plasma membrane-localized Ca²^+^ channels, Na^+^-Ca²^+^ exchangers, plasma membrane Ca²^+^-ATPases; mitochondrial Ca²^+^-H^+^ exchangers, and a repertoire of cytoplasmic Ca²^+^-binding proteins that act as Ca²^+^ buffers, fine-tuning cytoplasmic Ca²^+^ dynamics (Berridge et al., 2003). Viral infection, as an external stimulus, frequently disrupts intracellular Ca²^+^ homeostasis. RNA viruses such as HIV type 1, human T-lymphotropic virus type 1 (Ding et al., 2002), and hepatitis C virus (HCV) (Griffin et al., 2002), as well as DNA viruses including hepatitis B virus (Casciano and Bouchard, 2018) and herpes simplex virus types 1 and 2 (Cheshenko et al., 2003), have been shown to elevate cytosolic Ca^2+^ levels and perturb cellular Ca²^+^ homeostasis. This disruption is typically mediated by specific viral proteins that either facilitate the influx of extracellular Ca²^+^ into cells or induce the release of Ca²^+^ from intracellular stores. In this study, we found that VDCC blockers could significantly inhibit the increase in cytoplasmic Ca²^+^ concentration induced by DTMUV infection, but failed to completely abolish this effect. These results indicate that VDCCs are not the sole pathway mediating the elevation of cytoplasmic Ca²^+^ levels in DEFs upon DTMUV infection. Recent studies have reported distinct mechanisms of Ca²^+^ homeostasis disruption by closely related flaviviruses. For instance, WNV induces an influx of extracellular Ca²^+^ into infected cells by activating plasma membrane calcium channels (Scherbik and Brinton, 2010). In contrast, DENV disrupts intracellular Ca²^+^ homeostasis through two mechanistically distinct pathways: enhancement of plasma membrane permeability to Ca²^+^ and activation of the store-operated calcium entry (SOCE) pathway, which is governed by intracellular calcium stores (Dionicio et al., 2018). The aforementioned studies have demonstrated that in flavivirus-infected cellular models, both extracellular calcium influx and the release of calcium from intracellular stores disrupt cytoplasmic calcium homeostasis, resulting in elevated cytoplasmic Ca^2+^ concentrations. Therefore, it is reasonable to hypothesize that, beyond the VDCCs-mediated Ca²^+^ influx demonstrated in the present study, DTMUV infection may also trigger the release of Ca²^+^ from intracellular calcium stores via activating channels such as inositol 1,4,5-triphosphate receptors (IP_3_R) and ryanodine receptors (RyR) on the mitochondrial membrane, thereby elevating the cytoplasmic Ca²^+^ level. Further investigation is warranted to clarify these mechanisms in greater detail.

Additionally, it remains unclear how DTMUV infection activates L-type calcium channels on the plasma membrane. One possibility involves lipid rafts, which are cholesterol- and sphingolipid-rich microdomains that serve as unique signal transduction platforms. Multiple studies have demonstrated that lipid rafts and caveolar microdomains of the plasma membrane are enriched in proteins that regulate calcium signaling, including those that facilitate L-type calcium channel activation and calcium influx (Pani and Singh, 2009; Balijepalli et al., 2006). Furthermore, these lipid rafts are critically involved in the cellular entry of certain flaviviruses, including DENV and WNV (Osuna-Ramos et al., 2018). As a member of the Flavivirus genus, DTMUV is hypothesized to utilize lipid raft microdomains of the plasma membrane as a functional platform for aggregating key signaling molecules, thereby activating L-type calcium channels and mediating Ca²^+^ influx. Based on this proposed regulatory mechanism, subsequent studies should prioritize systematic, in-depth investigations to elucidate its molecular basis.

Current evidence suggests that Ca^2+^ plays a critical role in nearly every stage of the viral life cycle. Here, we demonstrated that the L-type calcium channel blocker diltiazem hydrochloride and the cytoplasmic calcium chelator BAPTA-AM reduce DTMUV yield by inhibiting the viral RNA replication step, without affecting viral entry or release. In previous studies on the interaction between cellular Ca^2+^ and viruses, pharmacologically reducing intracellular Ca^2+^ concentrations using calcium channel blockers or calcium chelators effectively inhibited viral RNA replication in Zika virus (ZIKV) (Bezemer et al., 2022), PRRSV (Manicassamy et al., 2023), porcine deltacoronavirus (PDCoV) (Bai et al., 2020), and respiratory syncytial virus (RSV) (Chen et al., 2024). Such findings are highly consistent with those of the present study. Within the Ca²^+^-mediated intracellular signal transduction network, AMPK is activated by CaMKK-II and functions as a key intracellular energy sensor, regulating critical physiological processes including cellular metabolism and autophagy (Salt et al., 2024; Herzig and Shaw, 2017). Therefore, from a mechanistic perspective, this study focused on AMPK as a central target for investigation. Our results revealed a coupling between the DTMUV infection-induced elevation of cytoplasmic Ca²^+^ and AMPK activation. Furthermore, treatment with an AMPK inhibitor significantly reduced DTMUV production by suppressing viral RNA replication—effects consistent with those observed upon treatment with VSCCs or a cytoplasmic Ca^2+^ chelator. These data indicate that cellular Ca^2+^ primarily regulates DTMUV replication via activation of the AMPK signaling pathway. This mechanism aligns with findings from studies of other viruses. For example, DEV infection increases cytoplasmic Ca²^+^ levels to activate the CaMKK-II–AMPK pathway and induce autophagy of DEFs; blocking this pathway with specific inhibitors significantly suppresses DEV replication (Yin et al., 2018). Similarly, PRRSV promotes its own replication via the same Ca²^+^–CaMKK-II–AMPK–autophagy axis (Manicassamy et al., 2023). Drawing on the rationale of the aforementioned studies, we investigated whether AMPK activation mediated by elevated cytoplasmic Ca²^+^ could induce autophagy in DEFs. We found that the expression level of microtubule-associated protein 1 light chain 3-II (LC3-II) in DEFs was significantly upregulated at 12 h post-infection with DTMUV. Interstingly, treatment of DEFs with BAPTA-AM and Compound C did not significantly alter the LC3-II expression level induced by DTMUV infection (**Supplementary Material**). Previously, Hu et al. confirmed that DTMUV infection can induce a complete autophagic response in DEFs by demonstrating the degradation dynamics of p62, the turnover of LC3-II throughout the infection cycle, and the formation of autolysosome-like vesicles observed via transmission electron microscopy (TEM) (Hu et al., 2020). In the present study, LC3-II was employed as the sole marker for autophagy detection, and experimental observations were restricted to a single time point at 12 hours post DTMUV infection. As a result, dynamic changes in autophagy throughout the entire course of DTMUV infection could not be fully captured. Thus, the available data are insufficient to elucidate whether autophagy modulates DTMUV replication via the calcium-AMPK signaling pathway, and the underlying molecular mechanisms merit further investigation. In addition, another potential mechanism is associated with oxidative stress, a biological state characterized by the disruption of the balance between the accumulation and clearance of intracellular reactive oxygen species (ROS). Accumulating evidence has confirmed that oxidative stress, Ca²^+^ signaling, AMPK activity, and autophagy form a highly interconnected regulatory network (Michalak et al., 2025). Among the Flavivirus genus, such as HCV, DENV, and Japanese encephalitis virus (JEV) have all been shown to induce ROS production, thereby triggering oxidative stress in infected cells (Korenaga et al., 2005; Guzman et al., 2014; Ghoshal et al., 2007). A close bidirectional interaction exists between ROS and Ca^2+^ signaling: on the one hand, ROS can modulate intracellular calcium signaling; on the other hand, Ca^2+^ signaling is a prerequisite for ROS generation (Görlach et al., 2015). For instance, studies have demonstrated that ROS facilitate calcium ion uptake in vascular smooth muscle cells via L-type and T-type voltage-gated calcium channels (Tabet et al., 2004); conversely, Ca²^+^ influx in neutrophils promotes ROS production by activating the recruitment of Ca²^+^-dependent S100A8/A9 proteins (Bréchard et al., 2013). Furthermore, in viral infection models, ROS can promote AMPK activation, thereby regulating autophagy and metabolism-related processes (Zhao et al., 2025). At the level of viral life cycle regulation, oxidative stress has been verified to modulate the capping of flaviviral RNA, which in turn enhances viral proliferation (Gullberg et al., 2015). Taken together, based on existing research findings and the data from the present study, we hypothesize that cellular oxidative stress may be involved in the regulation of viral replication via the Ca²^+^-AMPK axis induced by DTMUV infection, and this hypothesis warrants systematic verification in subsequent studies. Besides, it is worth noting that the Ca²^+^-CAMKK-II axis is not the sole pathway mediating the activation of AMPK during viral infection. As another upstream kinase of AMPK, liver kinase B1 (LKB1) also has the capacity to activate AMPK. For instance, the NS1 protein of dengue virus type 2 (DENV-2) could act as an assembly scaffold to facilitate the AMPK-LKB1 interaction, thereby inducing AMPK activation (Wu et al., 2023). Therefore, subsequent studies should investigate the role of LKB1 in AMPK activation induced by duck Tembusu virus (DTMUV) infection.

Flaviviruses typically establish an optimal replication environment by comprehensively modulating these metabolic processes of the host. On one hand, flavivirus infection can enhance the glycolytic pathway and pentose phosphate pathway of host cells, a phenomenon observed with viruses such as dengue virus DENV and JEV (Allonso et al., 2015; Li et al., 2021). The glycolysis process promoted by viral infection regulates multiple physiological processes, including increasing the reserves of adenosine triphosphate (ATP) and nucleotides to provide essential energy supply and precursor substances for material synthesis during viral replication. In addition, flavivirus replication requires the formation of invaginated structures on the endoplasmic reticulum membrane, known as replication compartments (RCs) (Gillespie et al., 2010). Flavivirus infection usually reshapes the host membrane system by promoting the synthesis of specific lipids to ensure the complete formation of RCs. For instance, WNV infection recruits 3-hydroxy-3-methylglutaryl-CoA reductase (HMGCR), a key enzyme in cholesterol biosynthesis, to RCs, thereby enhancing cholesterol synthesis and accumulation in the RC region. Inhibition of this process blocks WNV replication (Mackenzie et al., 2007). The non-structural protein 3 (NS3) of DENV relocalizes fatty acid synthase (FASN) to viral replication sites and promotes cellular fatty acid synthesis, thus facilitating the establishment of RCs (Heaton et al., 2010). Intracellular lipid droplets also serve as important targets for host metabolic regulation following flavivirus infection. Early in DENV infection, lipid droplet reabsorption into the endoplasmic reticulum can be observed, a process that may provide lipid raw materials for endoplasmic reticulum expansion and RC formation (Peña and Harris, 2012). In the late stage of infection, DENV induces lipophagy, a type of selective autophagy, through which lipids in lipid droplets are mobilized for β-oxidation and energy production (Heaton and Randall, 2010). As a central regulator of intracellular energy homeostasis, the activated AMPK modulates various cellular metabolic pathways, including lipid homeostasis maintenance and glycolysis, via phosphorylating downstream key targets (Herzig and Shaw, 2017). Based on the aforementioned findings regarding flaviviruses, we postulate that the activation of the AMPK pathway induced by DTMUV infection is likely involved in the reprogramming of host cellular metabolic programs, thereby establishing a favorable intracellular environment for viral replication. Further studies are required to elucidate the detailed molecular mechanism underlying this process.

In summary, our data demonstrate that DTMUV infection significantly disrupts Ca²^+^ homeostasis in host cells. Dysregulation of intracellular Ca²^+^ homeostasis activates the AMPK signaling pathway, which is a critical prerequisite for DTMUV to complete viral RNA replication. These findings provide a theoretical basis for elucidating the pathogenic mechanism of DTMUV and identifying novel antiviral targets.

## Data Availability

The original contributions presented in the study are included in the article/**Supplementary Material**. Further inquiries can be directed to the corresponding authors.
