# Supplementary material for: Disruption of cellular calcium homeostasis by duck Tembusu virus facilitates viral replication via AMPK pathway activation
Source: Front Cell Infect Microbiol. 2026 Feb 9;16:1743907. doi: 10.3389/fcimb.2026.1743907 (PMC12926480; doi:10.3389/fcimb.2026.1743907)
Supplement: Supplementary file 1 [file Table1.docx]

Supplementary Material

# Supplementary Figures and Tables

## Supplementary Figures


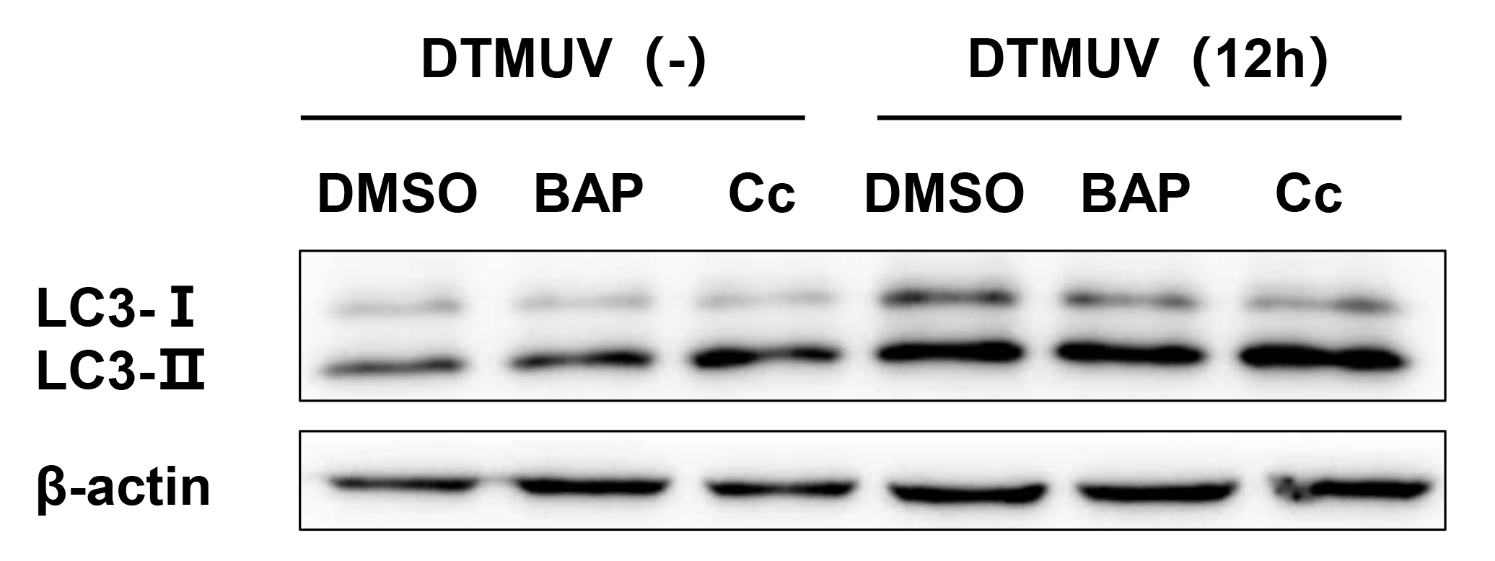


**Supplementary Figure 1.** **DTMUV Infection-induced LC3-** **II Expression Is Unaffected by Calcium Chelators or AMPK Inhibitors.** Immunoblotting analysis of LC3- II levels in DEFs treated with DMSO (control), BAPTA-AM (BAP; 25µM) and Compound C (Cc; 5µM) with or without DTMUV infection (MOI = 1).
